# Objections to Use of Carbolic Acid in Treatment of Piles

**Published:** 1878-06

**Authors:** 


OBJECTIONS TO USE OF CARBOLIC ACID IN TREATMENT OF PILES.—By J. M. Mathews, M.D., Louisville, Ky. Read before the Kentucky State Medical Society, April 4, 1878. (Reprint from the *American Medical Bi-Weekly*, April 27, 1878.)

